# 890. Environmental Air and Surface Sampling of Respiratory Viruses in Child Care Centers

**DOI:** 10.1093/ofid/ofad500.935

**Published:** 2023-11-27

**Authors:** Khalil Chedid, Peter Arts, Chris Blair, Andrew Hashikawa, Herek Clack, Krista Wigginton, Adam S Lauring, Linsey Marr, Aaron Prussin, Seema Lakdawala, Shweta Bansal, Anice Lowen, Emily T Martin

**Affiliations:** University of Michigan School of Public Health, 1317 Millbrook Trl, Michigan; University of Michigan, Ann Arbor, Michigan; University of Michigan, Ann Arbor, Michigan; University of Michigan, Ann Arbor, Michigan; University of Michigan, Ann Arbor, Michigan; University of Michigan, Ann Arbor, Michigan; University of Michigan, Ann Arbor, Michigan; Virginia Polytechnic Institute and State University, Blacksburg, Virginia; Virginia Polytechnic Institute and State University, Blacksburg, Virginia; Emory University, Atlanta, Georgia; Georgetown University, Washington D.C., District of Columbia; Emory University, Atlanta, Georgia; University of Michigan, Ann Arbor, Michigan

## Abstract

**Background:**

Approximately two-thirds of young children attend child care in the U.S. Child care attendees are at increased risk of acquiring respiratory infections. Determining transmission routes for respiratory viruses can aid in mitigating their spread. The study objective was to demonstrate the feasibility of sampling respiratory viruses from the child care environment and to determine their prevalence.

**Methods:**

Samples were collected from 9 child care centers (CCC) in Washtenaw County, MI from June 2022 to February 2023. Foam-tipped swabs were used for surface sampling. Aerosols were collected using TE-BC251 NIOSH Bioaerosol Cyclone samplers and a Series 110A Spot Sampler with 0.5% BSA in PBS. Total nucleic acids were extracted using the Chemagic 360 automated extraction platform. A probe-amplitude multiplex was used to measure multiple targets, including influenza A (IAV), respiratory syncytial virus (RSV), human rhinovirus (HRV), and severe acute respiratory syndrome coronavirus 2 (SARS-CoV-2), by RT-ddPCR.

**Results:**

A total of 839 surface samples, 150 samples each of large ( >4 µm), medium (1-4 µm), and small (< 1 µm) aerosols from the NIOSH sampler, and 19 Spot sampler samples were tested. In surface samples, HRV was most frequently detected (10/568 [1.8%]), but detection was rare for all viruses (Table 1). In NIOSH samples, RSV and HRV were most frequently detected (4/70 [5.7%] for RSV in large aerosols and 3/80 [3.8%], 1/80 [1.3%], and 3/80 [3.8%] for HRV in large, medium, and small aerosols). No viruses were detected in any Spot sampler samples. Sampling primarily occurred outside of peak IAV circulation in the community but overlapped with peak RSV and SARS-CoV-2 circulation. RSV was detected in aerosols from the same room on consecutive days in 1 CCC. HRV was detected in 3 rooms at 1 CCC in air and surface samples on one day. In another CCC, HRV was detected on 2 surfaces in 3 rooms one day and in aerosol samples in the same rooms the following day.

**Figure:**
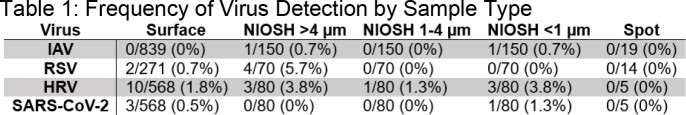

IAV: Influenza Virus A; RSV: Respiratory Syncytial Virus; HRV: Human Rhinovirus; SARS-CoV-2: Severe Acute Respiratory Syndrome Coronavirus 2; NIOSH: TE-BC251 NIOSH (National Institute for Occupational Safety and Health) Bioaerosol Cyclone

**Conclusion:**

Sampling respiratory viruses in the CCC environment is feasible. Their detection indicates the potential for environmental transmission. Expanded sampling during peak virus circulation can reveal routes through which respiratory viruses are spread in child care, informing infection prevention strategies to reduce their burden.

**Disclosures:**

**Khalil Chedid, MPH, MD, PHD**, Merck: Grant/Research Support **Andrew Hashikawa, MD**, Merck: Grant/Research Support **Herek Clack, PhD**, Taza Aya, Inc.: Ownership Interest **Adam S. Lauring, MD, PhD**, Roche: Advisor/Consultant|Sanofi: Advisor/Consultant **Emily T. Martin, PhD, MPH**, Merck: Grant/Research Support

